# Pathological tissue changes in brain tumors affect the pH‐sensitivity of the T1‐corrected apparent exchange dependent relaxation (AREX) of the amide protons

**DOI:** 10.1002/nbm.5285

**Published:** 2024-10-28

**Authors:** Eike Steidl, Elisabeth Neuhaus, Manoj Shrestha, Ralf Deichmann, Katharina Weber, Joachim P. Steinbach, Ulrich Pilatus, Elke Hattingen, Jan Rüdiger Schüre

**Affiliations:** ^1^ University Hospital, Institute of Neuroradiology Goethe University Frankfurt Frankfurt Germany; ^2^ University Hospital, University Cancer Center (UCT) Goethe University Frankfurt Frankfurt Germany; ^3^ Brain Imaging Center Goethe University Frankfurt Frankfurt Germany; ^4^ University Hospital, Neurological Institute (Edinger Institute) Goethe University Frankfurt Frankfurt Germany; ^5^ Frankfurt Cancer Institute (FCI) Goethe University Frankfurt Frankfurt Germany; ^6^ German Cancer Consortium (DKTK) Partner Site Frankfurt, and German Cancer Research Center (DKFZ) Heidelberg Germany; ^7^ University Hospital, Institute of Neurooncology Goethe University Frankfurt Frankfurt Germany; ^8^ Institute of Neuroradiology, University Clinic Erlangen Friedrich‐Alexander‐Universität Erlangen‐Nürnberg Erlangen Germany

**Keywords:** APT‐CEST, AREX, MR‐spectroscopy, pH, T1‐relaxometry

## Abstract

Measuring the intracellular pH (pHi) is of interest for brain tumor diagnostics. Common metrics of CEST imaging like the amide proton transfer‐weighted (APTw) MTR_asym_ are pHi sensitive and allow differentiating malignant tumor from healthy tissue. Yet, the image contrast also depends on additional magnetization transfer effects and T1. In contrast, the apparent exchange‐dependent relaxation (AREX) provides a T1 corrected exchange rate of the amide protons. As AREX still depends on amide proton density, its pHi sensitivity remains ambiguous. Hence, we conducted this study to assess the influence of pathologic tissue changes on the pHi sensitivity of AREX in vivo. Patients with newly diagnosed intra‐axial brain tumors were prospectively recruited and underwent conventional MRI, quantitative T1 relaxometry, APT‐CEST and ^31^P‐MRS on a 3T MRI scanner. Tumors were segmented into contrast‐enhancing tumor (CE), surrounding T2 hyperintensity (T2‐H) and contralateral normal appearing white matter (CNAWM). T1 mapping and APT‐CEST metrics were correlated with ^31^P‐MRS‐derived pHi maps (Pearson's correlation). Without differentiating tissue subtypes, pHi did not only correlate significantly with MTR_asym_ (*r* = 0.46) but also with T1 (*r* = 0.49). Conversely, AREX only correlated poorly with pHi (*r* = 0.17). Analyzing different tissue subtypes separately revealed a tissue dependency of the pHi sensitivity of AREX with a significant correlation (*r* = 0.6) in CNAWM and no correlation in T2‐H or CE (*r* = −0.11/−0.24). CE showed significantly increased MTR_asym_, pHi, and T1 compared with CNAWM (*p* < 0.001). In our study, the pHi sensitivity of AREX was limited to CNAWM. The lack of sensitivity in CE and T2‐H is probably attributable to altered amide and water proton concentrations in these tissues. Conversely, the correlation of pHi with MTR_asym_ may be explained by the coincidental contrast increase through increased T1 and amide proton density. Therefore, limited structural deviations from CNAWM might be a perquisite for the use of CEST contrasts as pHi‐marker.

## Graphical abstract

Without differentiating tissue subtypes, pHi correlated significantly with MTR_asym_ and T1 but not with the T1‐corrected AREX (*r* = 0.46/0.49/0.17). Analyzing tissue subtypes separately revealed a tissue dependency of the pHi sensitivity of AREX with a significant correlation in contralateral white matter and no correlation in T2‐hyperintensity/contrast enhancing tumor (*r* = 0.6/−0.11/−0.24), probably attributable to altered amide and water proton concentrations. Therefore, limited structural tissue changes might be a perquisite for the use of CEST contrasts as pHi‐marker.

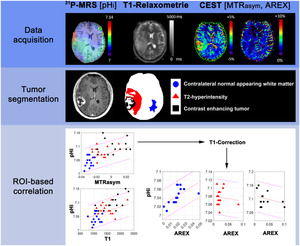

**Abbreviations**
APT‐CEST: amide proton transfer chemical exchange saturation transfer
AREX: apparent exchange‐dependent relaxation
CE: contrast‐enhancing tumor tissue
CNAWM: contralateral normal‐appearing white matter
FLAIR: fluid‐attenuated inversion recovery
FoV: field of view
IDH: isocitrate dehydrogenase
MRS: MR spectroscopy
MTR_asym_
: APT‐weighted asymmetric magnetisation transfer ratio
MTR_Rex_
: magnetisation transfer ratio from the relaxation rate
MT: magnetisation transfer
N: necrosis
NOE: nuclear overhauser effect
pHi: intracellular pH
ROI: region of interest
ssMT: semi‐solid magnetisation transfer
T2‐H: surrounding T2‐hyperintensity
VFA: variable flip angle

## 1 INTRODUCTION

Malignant brain tumors are characterized by distinct changes in their metabolic pathways that are believed to facilitate cell proliferation and invasion as well as tumor immune escape.^1^, ^2^ Some of these metabolic changes are detectable in vivo via 3 Tesla MRI. Methods for metabolic MR measurements are ^1^H‐ and ^31^P‐MR spectroscopy (MRS) as well as the more recently established amide proton transfer–chemical exchange saturation transfer (APT‐CEST) imaging. These methods usually allow to noninvasively differentiate tumor tissue from healthy brain tissue.^3^, ^4^ Furthermore, metabolic MR measurements have been used for therapy monitoring and to identify predictive biomarkers,^5^ as well as to obtain mechanistic insights into the in‐vivo effects of novel treatments like antiangiogenic therapy.^6^, ^7^ Moreover, the ability to monitor metabolic changes in vivo is of interest in the context of innovative therapeutic approaches like tumor specific vaccines or cell therapies.^8^, ^9^

^31^P‐MR spectroscopy (^31^P‐MRS) is able to measure intracellular pH (pHi), a parameter that is critically relevant in the homeostasis of malignant tumors. For example, the increased pHi levels in glioblastoma favor glycolysis.^10^ At the same time the acidification due to the increased amounts of Lac and H+ is compensated via an upregulation of the carbonic anhydrase^11^ or transmembrane transporters like the Na+/H+ exchanger^12^ and monocarboxylate transporters.^13^ The ensuing acidification of the tumor microenvironment again promotes cell invasion and drug resistances.^14^ Hence, noninvasive monitoring of pH levels should provide essential information about the malignant potential of a brain tumor. Yet, pH mapping via ^31^P‐MRS has major limitations such as long scanning times, low spatial resolution and the need for dedicated MR coils. The amide proton exchange rate does also depend on the pH of the environment. Thus, APT‐CEST imaging has been discussed as an alternative method for measuring pHi.^15^ It would overcome the specific limitations of ^31^P‐MRS and is applicable in a clinical setting with ^1^H coils.

Over the last years, the growing interest in APT‐CEST for brain tumor imaging has spurred the development of CEST protocols on clinical scanners, which also include basic data analysis. It has been shown that the commonly used APT‐weighted asymmetric magnetization transfer ratio (MTR_asym_) correlates well with pHi obtained by ^31^P‐MRS.^16^, ^17^ However, this metric is sensitive to T1 and combines several magnetization transfer effects. Thus, the contribution from pHi‐related changes in APT‐CEST is not known. Without this knowledge, the direct link between pHi and CEST contrast is missing, possibly rendering the observed correlation as just coincidence and thereby limiting its role as reliable pH indicator. In the presented approach, we studied the pHi dependency of the apparent exchange dependent relaxation (AREX) of the amide protons in a cohort of patients with brain tumors. The increased pHi in tumors was determined with ^31^P‐MRS and correlated with AREX, which presents a T1‐corrected measure for the APT‐CEST component in the Z‐spectrum, eliminating the role of other MT components such as the semi‐solid MT (ssMT) and the nuclear overhauser effect (NOE).

We conducted this prospective study to correlate common APT‐CEST metrics with ^31^P‐MRS pHi measurements and to evaluate the impact of T1 correction on the pH sensitivity of APT‐CEST in a clinical setting at 3 T.

## 2 MATERIALS AND METHODS

### 2.1 Patient cohort

This prospective study was approved by the ethics committee of our university hospital (E8/20, E159/18). Patients who met the following inclusion criteria were prospectively recruited.

- MRI‐based diagnosis of an intra‐axial tumor
- No previous anti‐tumor therapy
- Sufficient clinical condition for additional MRI examinations
- At least 18 years old
- Written and informed consent could be obtained.

### 2.2 Data acquisition

All patients underwent diagnostic MRI scans at a field strength of 3 T (MAGNETOM Skyra‐fit, Siemens Healthineers, Erlangen, Germany) including T2‐weighted imaging, fluid‐attenuated inversion recovery (FLAIR), and T1‐weighted imaging with and without contrast enhancement. Additional data sets for CEST, quantitative T1 mapping, and ^31^P‐MRS were acquired on a 3‐T whole‐body MRI system (MAGNETOM Prisma, Siemens Healthineers, Erlangen, Germany) in separate sessions no later than 3 days after the initial diagnostic MRI. The detailed image acquisition parameters for the study sequences are listed below.

#### 2.2.1 Quantitative T1 relaxometry (T1)

Three‐dimensional GRE datasets were acquired according to the variable flip angle (VFA) method^18^ with a voxel size = 1 mm^3^, FoV = 256 × 224 × 160 mm^3^, TE = 6.7 ms, TR = 16.4 ms, bandwidth = 222 Hz/Pixel, FA1 = 4°, FA2 = 24°. Further corrections for B1 inhomogeneities were performed via the acquisition of two additional GRE datasets with and without magnetization preparation via a 45° RF pulse,^19^ while B0 correction was based on a 2D multislice dual‐echo GRE sequence with export of magnitude and phase data. Acquisition parameters are as follows: FoV = 256 × 224 × 160 mm^3^, matrix size = 64 × 56, number of slices and spatial resolution of 4 × 4 mm^2^ identical for B0 and B1 mapping.

#### 2.2.2 APT‐CEST

Data were acquired using a fast CEST‐EPI sequence with a voxel size of 3 × 3 × 4 mm^3^, 16 slices, FoV = 256 × 256 mm^2^, bandwidth = 2298 HZ/Pixel, TE1/TE2 = 22.08 ms/23.28 ms, and TR = 8000 ms. After bringing the initial magnetization into the steady‐state by using a presaturation module of 16 rectangular dummy RF pulses, the main saturation module uses just one rectangular RF pulse to stay in the steady state followed by the EPI readout. The pulse shape for saturation compromises an RF duration of 250 ms, followed by a 250‐ms delay and B_1,max_ = 1 μT (B_1,rmse_ ≈ 0.7μT). CEST data were acquired in the spectral range from +8 ppm to −8 ppm (decrement 0.5 ppm) and from ±3 ppm to ±4 ppm (increment 0.1 ppm). A comprehensive description of the multislice CEST measurement can be found in the literature.^20^

#### 2.2.3 ^31^P‐MRS

Spectroscopic data were acquired by using a 3D FID‐CSI with a voxel size of 30 × 30 × 25 mm^3^, FA = 60°, FoV = 240 × 240 × 200 mm^3^, bandwidth = 2000 Hz/Pixel, TR/TE = 2000 ms /2.3 ms, and 10‐fold averaging. An anatomical reference scan was further included for planning the spectroscopic measurement. Therefore, a 3D T1‐w MPRAGE sequence was used with 1‐mm isotropic resolution, FoV = 256 × 256 × 192 mm^2^, TI/TR/TE = 900 ms/1900 ms/3.2 ms.

### 2.3 Preprocessing

Data preprocessing and analysis were performed in MATLAB (The Mathworks, Natick, MA, USA) in combination with the software package FSL (version 5.0.9) and jMRUI for evaluating phosphorus data.

Spectra from ^31^P MRS were manually phase shifted, and the signal of phosphocreatine (PCr) was adjusted as reference signal to 0 ppm, before the spectra were analyzed with AMARES (Advanced Method for Accurate, Robust and Efficient Spectral Fitting) in the time domain of the FID signal.^21^ Prior knowledge of several exponentially decaying sinusoidal curves with fitted initial values for the determination of the frequency offsets and widths of the individual spectra was used for fitting the metabolite signals. ^31^P‐pHi was calculated according to the spectral distance *δ*_obs_ between the signals of PCr and inorganic phosphate (Pi) by using the modified Henderson–Hasselbach equation^22^ (Equation 1).

(1) $$\mathrm{pHi}=\mathrm{pka}+\log\;\left(\delta_{\mathrm{obs}}-3.29/5.68-\delta_{\mathrm{obs}}\right)$$

For obtaining quantitative T1 data, the two acquired GRE datasets were co‐registered via FLIRT.^20^ Further preprocessing steps included skull removal and tissue segmentation via BET^23^ and FAST.^24^ The further process including corrections for insufficient transverse RF‐spoiling and B1 inhomogeneities and the final T1 calculation were performed as described in the literature.^21^ Quantitative T1 maps were then co‐registered with the CEST data.

For APT‐CEST preprocessing, a voxel‐based correction for lipid artifacts was performed according to Dixon.^25^ In detail, magnitude and phase data for each frequency offset were acquired at the echo times TE1 and TE2, which differed by 1.2 ms, thus yielding a 180° phase shift allowing to distinguish the signals of water and fat. Subsequently, all CEST data were corrected for motion artifacts via FLIRT^20^ before calculating Z‐spectra for each voxel. To correct for B0 inhomogeneities, Z‐spectra were fitted voxel‐wise in the spectral range from −1 to +1 ppm with an inverted Lorentzian function to determine the minimum corresponding to 0 ppm^26^ and then shifted according to the determined B0 drift. Lorentz fitting was then used to obtain a 4‐pool system (Zref) including the aliphatic NOE, ssMT, amide, and water proton pools. Additionally, a 3‐pool model (Zlab), which excludes the amide proton pool, was also applied.

For the evaluation of the different APT‐CEST metrics, the APT‐weighted MTR_asym_ was calculated according to the literature^15^ (Equation 2):

(2) $${\mathrm{MTR}}_{\text{asym}}\;\left(3.5\;\mathrm{ppm}\right)=Z\left(-3.5\;\mathrm{ppm}\right)-Z\left(+3.5\;\mathrm{ppm}\right)$$

As the APTw image contrast is affected by the overlying ssMT and aliphatic NOE at −3.5 ppm, the isolation of the amide signal was achieved by using Zlab and Zref for the calculation of the magnetization transfer ratio from the relaxation rate (MTR_Rex_)^27^ of the exchanging protons at 3.5 ppm.

(3) $${\mathrm{MTR}}_{\mathrm{Rex}}\left(3.5\;\mathrm{ppm}\right)=\left(1/\text{Zlab}\left(3.5\;\mathrm{ppm}\right)\right)-\left(1/\text{Zref}\left(3.5\;\mathrm{ppm}\right)\right)$$

As MTR_Rex_(3.5 ppm) describes the relaxation rate of the exchanging protons (Rex) at this specific frequency offset, but scaled by the relaxation rate of water, a T1 correction was performed according to Equation (4) via the co‐registered quantitative T1 datasets, yielding the so‐called apparent exchange dependent relaxation (AREX).^27^

(4) $$\text{AREX}\;\left(3.5\;\mathrm{ppm}\right)={\mathrm{MTR}}_{\mathrm{Rex}}\left(3.5\;\mathrm{ppm}\right)/\text{T1}=R_{\mathrm{ex}}$$

This term now describes the pH‐dependent relaxation rate ksw of the exchanging amide protons at 3.5 ppm with water protons as well as their ratio fb under the assumption of full saturation (*α* ≈ 1). This can also be expressed as

(5) $$R_{\mathrm{ex}}=\mathrm{ksw}*\mathrm{fb}$$

After calculating all APT‐CEST metrics via the equations given above, the CEST maps were co‐registered with FSL onto the MPRAGE dataset that had been used for planning the phosphorus spectroscopic measurement.

### 2.4 Tumor segmentation

Based on the diagnostic MRI scans, all datasets were automatically segmented into contrast‐enhancing tumor tissue (CE), necrosis (N), and surrounding T2‐hyperintensity (T2‐H, corresponding to edema and/or nonenhancing tumor) using the freely available software BraTS Toolkit.^28^ The region of interest (ROI) containing necrosis was not included in the analysis. All segmentations were manually checked and, if necessary, corrected by an experienced radiologist. Also, a ROI for contralateral normal‐appearing white matter (CNAWM) was defined manually.

### 2.5 Statistics

To address the question to what extent APT‐CEST imaging reflects spectroscopic pHi, a regression analysis via Pearson's correlation was performed using the median values of the tissue segmentation ROIs (CE, T2‐H, CNAWM). The spectroscopic pHi was compared with the three different APT‐CEST metrics and tested for significance using the paired *T* test. Significant differences between tissue types were determined for pHi and the T1‐corrected APT‐CEST effect via ANOVA. To outline the differences between the different tissue types, the pHi, T1 values, and APT‐CEST contrasts were compared for the different tissue segmentations.

## 3 RESULTS

We recruited 17 patients (median age 60 years, range 38–67 years; 14 male [82%]). Ten patients were diagnosed with glioblastomas (WHO grade 4, isocitrate dehydrogenase [IDH] wildtype), four with metastasis (primary: two malignant melanoma, two nonsmall cellular lung cancer), two with astrocytoma (WHO grade 3, IDH mutant), and one with a primary central nervous system lymphoma.

**FIGURE 1:**
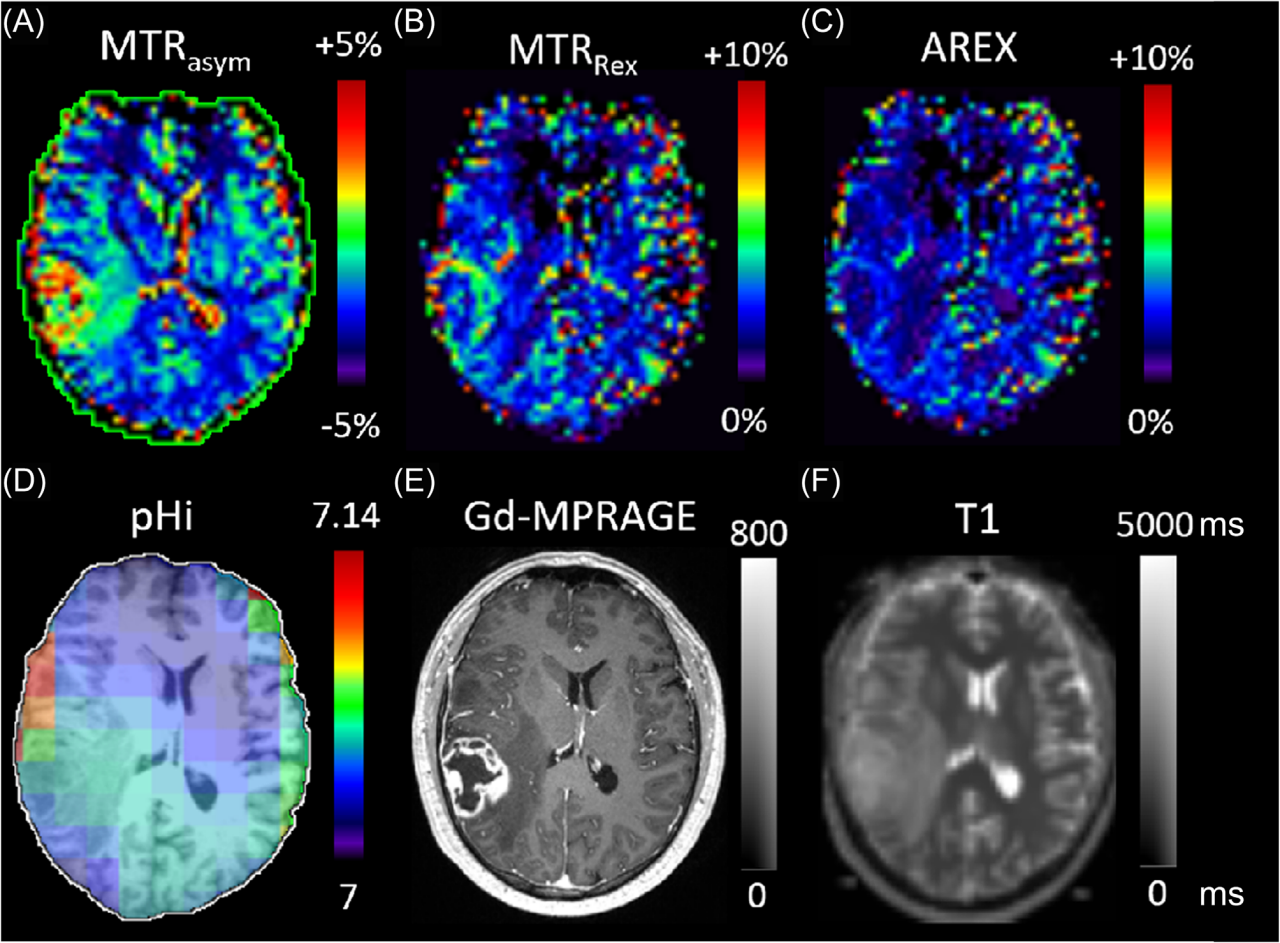
Representative data from a patient with a glioblastoma in the right temporoparietal region. Color maps for the APT‐CEST contrast at 3.5 ppm ((a) MTR_asym_, (b) MTR_Rex_, (c) AREX) and the intracellular pH levels measured by ^31^P‐MRS ((d) pHi). (e) shows the corresponding slice of a contrast enhanced T1 MPRAGE as anatomical reference and (f) the quantitative T1 map (T1). While various magnetization transfer effects in MTR_asym_ lead to high image contrast of the tumor, the Lorentzian fitted amide pool corrections leading to MTR_Rex_ decrease the contrast especially in the necrotic center. The ring‐shaped, contrast enhancing tumor structure is still recognizable after T1 correction resulting in AREX, but the intensity is further decreased. Even though limited in resolution, the pH_i_ map depicts increased values in the tumor region, gradually decreasing towards the contralateral, normal appearing tissue. Adjacent to the brain surface or skull artifacts are visible.

For illustration, Figure 1 shows representative data from a patient with a glioblastoma. The three evaluated APT‐CEST metrics are shown in the top row, the lower row displays pHi, MPRAGE with contrast agent, and T1. It is noteworthy how the image contrast of contrast enhancing tumor tissue, which is also visible in pHi and T1 (Figure 1D,F), is strongest in MTR_asym_ and becomes less apparent with increasing number of correction methods applied (Figure 1A–C).

**FIGURE 2:**
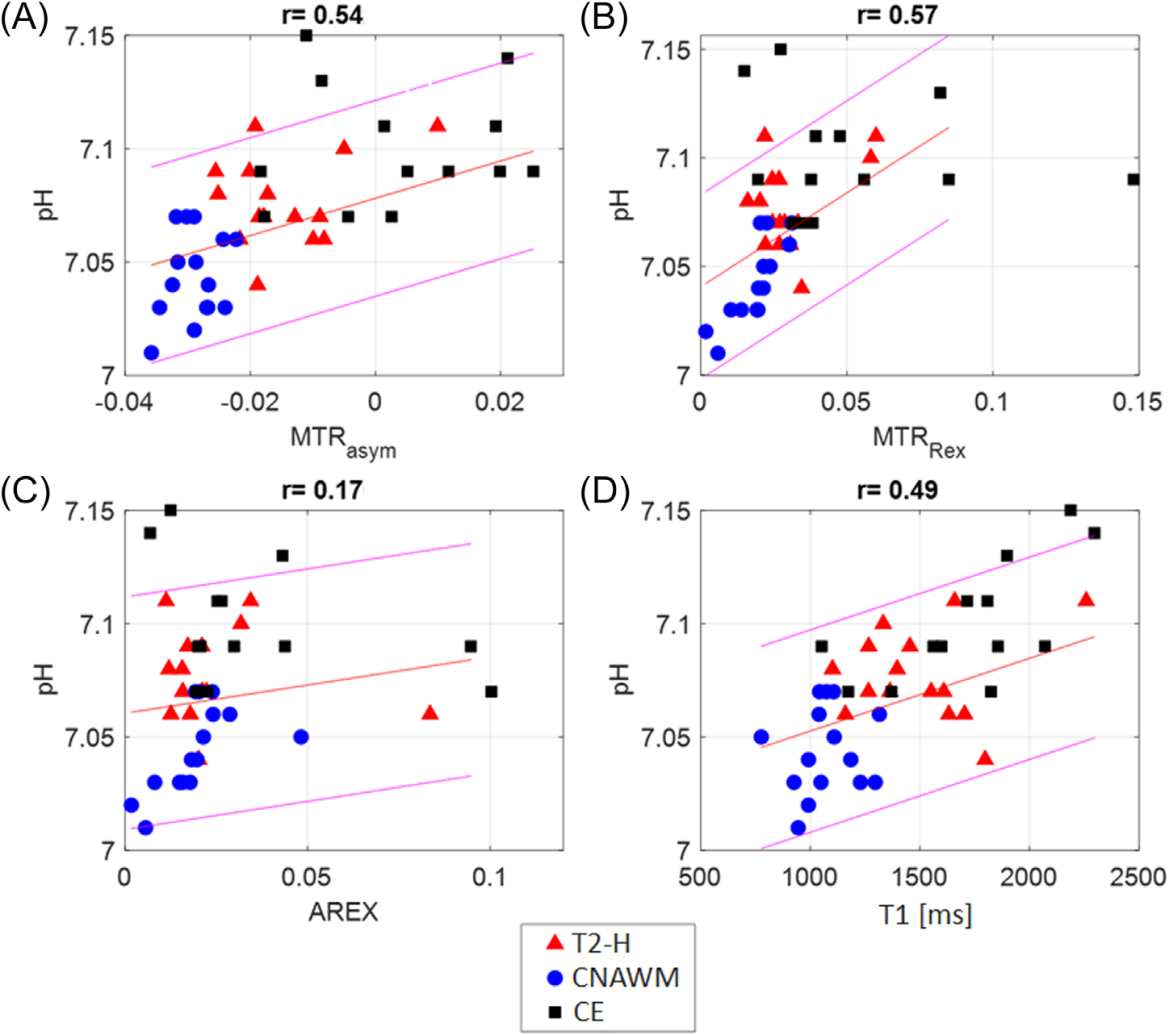
Scatterplots with linear regression line, 95% confidence interval and *r* values from Pearson's correlation for the correlation of the intracellular pH levels measured by ^31^P‐MRS (pH_i_) with MTR_asym_ (a), MTR_Rex_ (b), AREX (c), and the T1 relaxation time (T1, in milliseconds, (d)). Red triangles represent data points derived from T2 hyperintense (T2‐H) tissue, blue dots from contralateral normal appearing white matter (CNAWM), and black squares represent data from contrast enhancing tissue (CE).

To gain a better insight into the correlation between pH_i_ and APT‐CEST, respective data from the different tissue segmentation ROIs (CE, T2‐H, CNAWM) were collected and pH_i_ was plotted against each evaluated parameter. There was a significant correlation between pH_i_ and T1 (*r* = 0.49, *p* < 0.001; Figure 2D). Regarding the different CEST metrics, the overall correlation with pH_i_ was significant for MTR_asym_ (*r* = 0.54, *p* < 0.001; Figure 2A) and MTR_Rex_ (*r* = 0.58, *p* < 0.001; Figure 2B), while AREX did not correlate with pH_i_ (*r* = 0.17, *p* = 0.28; Figure 2C).

**FIGURE 3:**
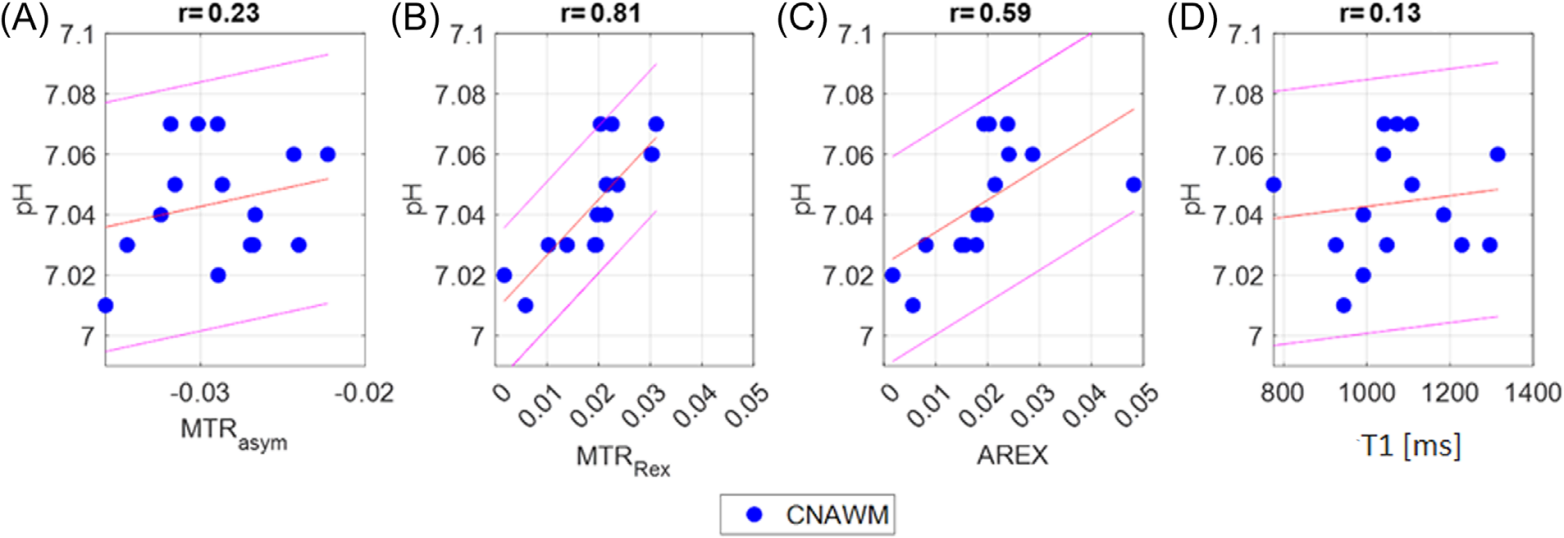
Scatter plots with linear regression line, 95% confidence interval and *r* values from Pearson's correlation for the correlation of the intracellular pH levels measured by ^31^P‐MRS (pH_i_) and APT‐CEST contrasts at 3.5 ppm for (a) MTR_asym_, (b) MTR_Rex_, (c) AREX, and the quantitative T1 (T1, in milliseconds). Only data derived from the contralateral normal appearing white matter (cNAWN) are shown.

**FIGURE 4:**
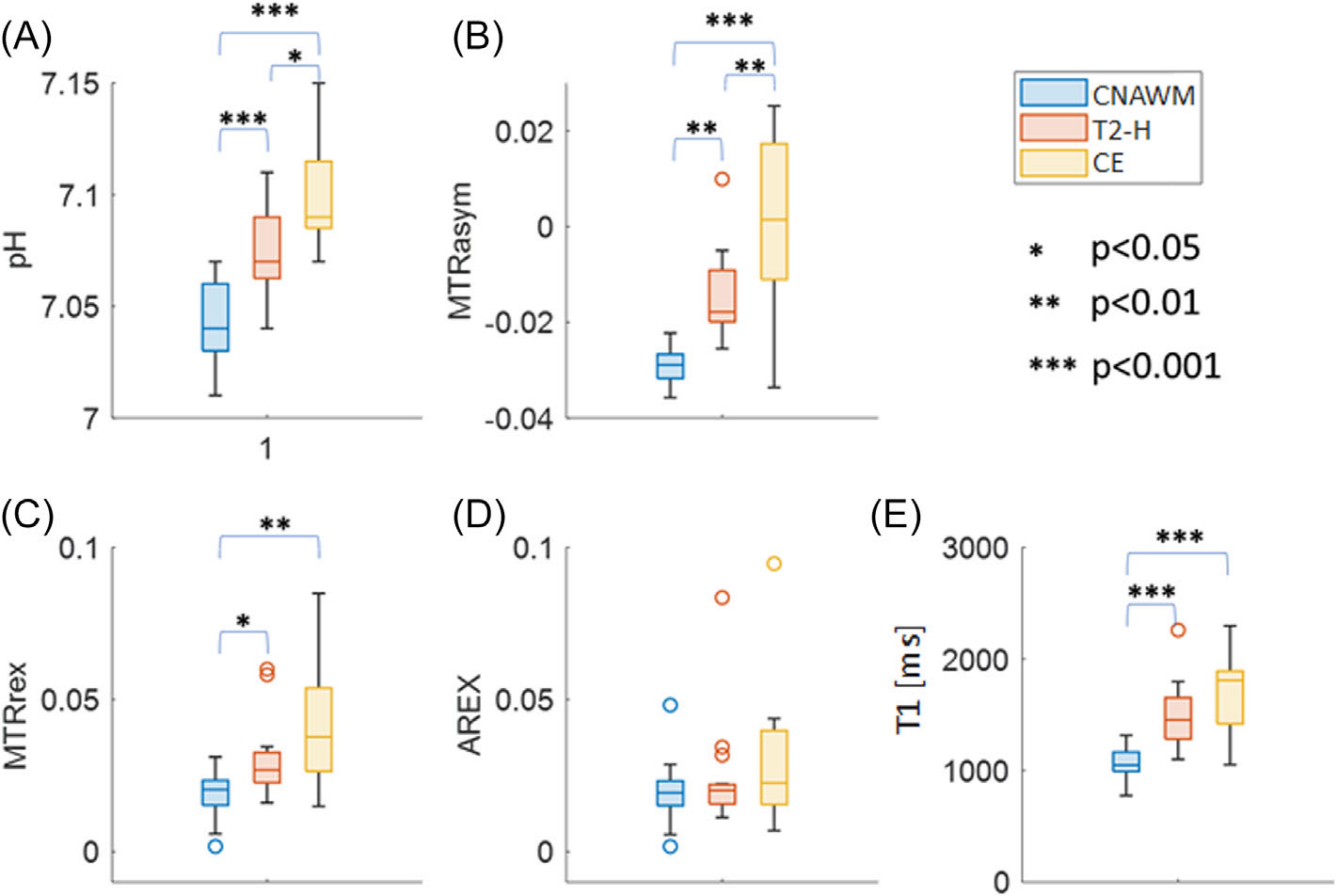
Boxplots of the different tissue types (contralateral normal appearing white matter (CNAWM, blue), T2 hyperintense tissue (T2H, red) and contrast enhancing tissue (CE, yellow)) for intracellular pH levels measured by ^31^P‐MRS (pH_i_, (a)), the different APT‐CEST contrasts at 3.5 ppm (MTR_asym_ (b), MTR_Rex_ (c), AREX (d)) and the quantitative T1 (T1, in milliseconds, (e)). Significant differences are indicated by *.

As the results especially in tumor tissue appeared to be heterogeneous, we analyzed the correlations of all CEST metrics and pH_i_ separately for CNAWM as shown in Figure 3. While the T1 values showed a significant correlation with pH_i_ when all ROIs were combined, no correlation could be detected when evaluating CNAWM alone (*r* = 0.13, *p* = 0.63; Figure 3D). Similarly, no significant correlation could be observed for MTR_asym_ (Figure 3A; *r* = 0.24, *p* = 0.39). Yet, the isolation of the amide peak via MTR_Rex_ revealed a strong correlation with the spectroscopic pH_i_ (Figure 4B; *r* = 0.82, *p* < 0.001), exceeding the correlation of MTR_Rex_ and pH_i_ in the combination of all tissue ROIs. Likewise, a significant relationship of AREX and pH_i_ could be observed in CNAWM (Figure 4C; *r* = 0.60, *p* = 0.018) that was not evident in the combination of all ROIs. In line with these findings, the additional analysis of the separate relations of AREX and pH_i_ in T2‐H and CE revealed no correlations as shown in the Supporting Information (Figure S1a,b; *r* = −0.11/−0.24, *p* = 0.69/0.45).

To characterize the potential differences in the three tissues types, a boxplot analysis was performed for all measured parameters (Figure 4). In contrast‐enhanced tissue, the pH_i_ (Figure 4A) was significantly higher than in CNAWM (*p* < 0.001). Also, CE showed significantly increased MTR_asym_ (*p* < 0.001; Figure 4B) and MTR_Rex_ (*p* < 0.01, Figure 4C) contrast as well as longer T1 (*p* < 0.001; Figure 4E) compared with CNAWM.

In T2‐H, the pH_i_ was also significantly higher than in CNAWM (*p* < 0.001; Figure 4A). We further found significantly elevated values for MTR_asym_ (*p* < 0.01; Figure 4B) and T1 (*p* < 0.001; Figure 4E) in T2‐H compared with CNAWM.

When comparing CE and T2‐H against each other, pH_i_ and MTR_asym_ were significantly increased in CE, while this effect was not visible in MTR_Rex_ and T1. Also, for AREX, no significant alternations between the tissues were found.

**FIGURE 5:**
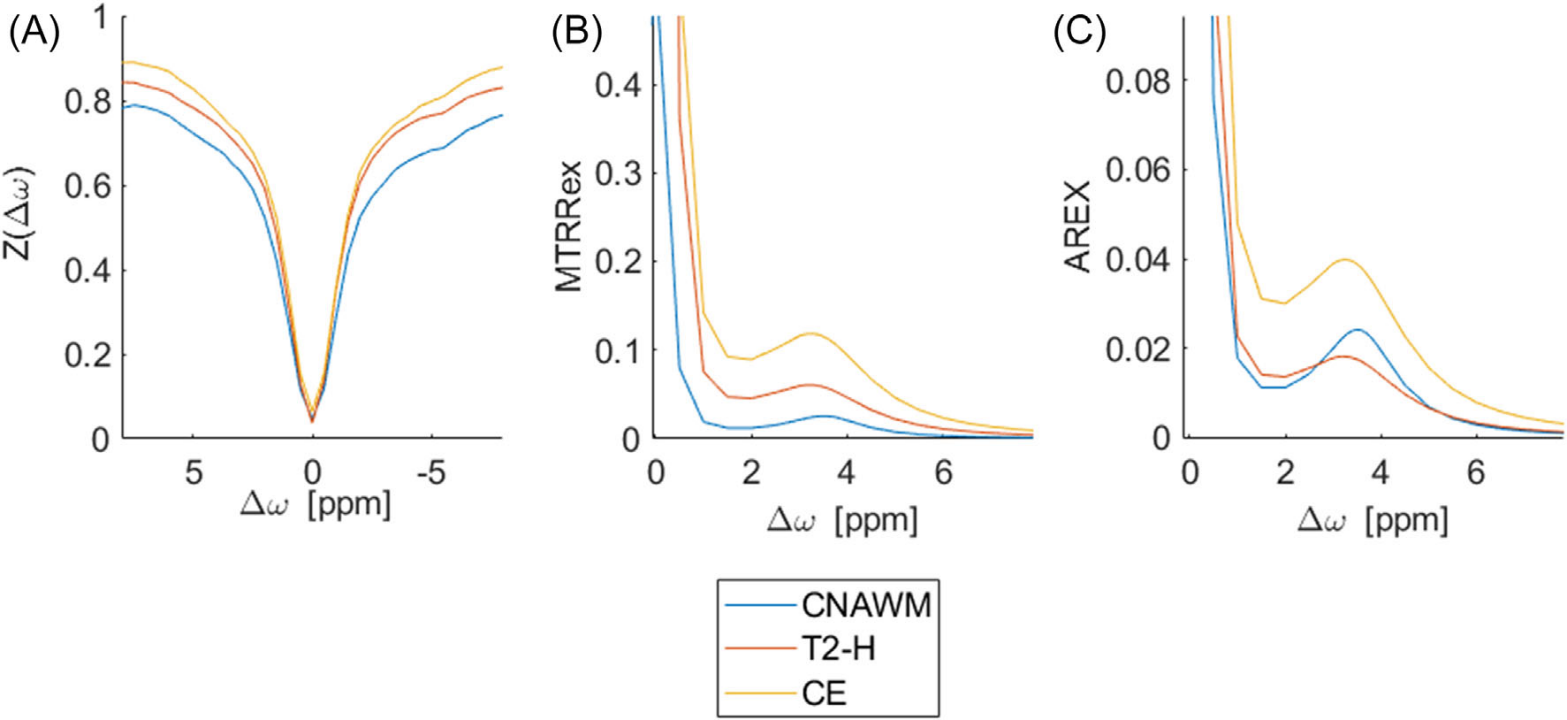
In (a) exemplary Z‐spectra acquired in the three different ROIs (contralateral normal appearing white matter (CNAWM), surrounding T2 hyperintensity (T2‐H), contrast enhancing tumor (CE)) are shown. Panel (b) displays the calculated MTR_Rex_ and (c) AREX in the range from 0 to 8 ppm. As visible in (a), the ROIs with higher water and possibly blood components such as edema (T2‐H) and contrast‐enhancing tissue (CE) generally display a raised amplitude of the Z‐spectrum. When observing MTR_Rex_, the signal of the different tissue types is still dependent on T1, which explains the higher signal intensities within T2‐H and CE tissue compared with CNAWM. When correcting for T1 according to Equations (4) and (5), the signal is now solely dependent on pHi and the proton fraction at Δ*ω*.

Figure 5 displays exemplary Z‐spectra (Figure 5A) and corresponding spectra for MTR_Rex_ and AREX (Figure 5B,C) in the three ROI types separately, demonstrating a signal increase in T2‐H and CE.

## 4 DISCUSSION

We examined 17 patients with intra‐axial tumors and correlated different CEST metrics with ^31^P‐MRS‐derived pH_i_ and T1 values in pathologic and contralateral normal appearing tissue. As a main finding, the correlation of both the different CEST metrics and T1 with pH_i_ showed a marked dependence on the tissue type.

Data evaluation without discrimination of the different tissue subtypes yielded a significant correlation between T1 and pH_i_ (*r* = 0.5, Figure 2D). Yet, to our knowledge, there is no direct physical connection between longitudinal relaxation and pH_i_.^29^ Hence, our statistical finding is likely a correlation without causation caused by a pH_i_ increase in CE and T2‐H due to metabolic changes^30^, ^31^ and a T1 increase mostly due to an increased water and blood content.^32^, ^33^ The exemplary z‐spectra for CE, T2‐H, and CNAWM in Figure 5A depict the different signal intensities, likely caused by the combination of the above mentioned effects. Consequently, it can be expected that T1 and the pH_i_ sensitive MTR_asym_ also correlate well with pH_i_ (Figure 2A). One possibility to overcome this issue in future MTR_asym_ analysis is to use fluid suppression, as recently suggested in the literature.^34^, ^35^

The same effect was visible after the correction for ssMT and aliphatic NOE (MTR_Rex_), even though both MT effects likely also benefit a high tumor contrast due to a changing tissue structure^36^ (Figure 5B). On the contrary, when additionally correcting for T1, the resulting AREX did not correlate significantly with pH_i_ anymore (Figure 2C). This could be attributed to the fact that the image contrast is not solely influenced by pH changes, as demonstrated in vitro^27^, ^37^, ^38^ but is also dependent on the concentration of proteins and peptides.^37^ Fittingly, in the positive spectrum shown in Figure 5C, a persisting signal increase of CE compared with T2‐H and NAWM can be observed.

To further investigate our finding, we analyzed the three different tissue ROIs separately. When specifically analyzing just CNAWM, both T1 and MTR_asym_ lost their correlation with pHi (Figure 3A,D). Noteworthy, the correction for ssMT and aliphatic NOE led to a significant correlation between MTR_Rex_ and pH_i_ (Figure 3B). This correlation was still present after T1 correction, meaning AREX also correlated significantly with pH_i_ in CNAWM (Figure 3C). This result seems to be in line with the theoretic considerations and can be replicated in vitro using phantom tubes with stable proton and amide concentrations but increasing T1 and pH (Figure S2). As per definition, CNAWM does not show any signal deviations on diagnostic MRI; it likely constitutes a relatively stable environment without major deviations in either water content (no visible edema) or amide proton density (among others no visibly increased cell density). As a result, it can be assumed that the ratio fb of the amide and water protons is stable, thus more closely resembling the controlled in vitro environment.

Consequently, changes in AREX should reflect the pH sensitive exchange rate ksw (Equation 5). The observed, minor variance of pH_i_ in CNAWM in our cohort is possibly caused by slight increases of pH_i_ in several glioblastoma patients. This is consistent with previous glioma studies, in which the pH_i_ value in CNAWM was slightly higher compared with a healthy control group and was believed to be associated with microscopic tumor infiltration.^16^, ^30^

In contrast, in the differential analysis of both CE and T2‐H, AREX did not show any correlation with pH_i_ (Figure S1). As shown in Figure 4, the dispersion of the pH_i_ and T1 values is much higher in CE and T2‐H than in CNAWM. This is expected, as the intensity of edema likely differs in between patients and different tumors will likely display differences in their metabolism and cell density. Also, especially malignant gliomas often show an increased blood volume,^39^ and as previously discussed,^40^ the high protein content of blood could significantly contribute to the CEST signal. This would be in line with the T1 values in CE being relatively close to the T1 of blood^41^ in our cohort. Thus, concerning CEST measurements, not only the pH dependent exchange rate ksw but also the proton ratio fb varies in CE and T2‐H. This implies that AREX will likely not reflect pH_i_ changes as long as pathological variations are involved that alter the water and mobile protein/peptide concentration as also discussed in the literature.^42^, ^43^, ^44^ As a consequence, measurements of AREX in pathologically altered tissue should be interpreted with caution.

To further isolate specific biologic processes like pH_i_ changes, new methods are needed to counteract different proton fractions in CEST experiments. Yet, the limited signal intensities at 3 T currently pose a limitation for the combination of several correction methods. Further, a limitation in our study is the number of patients, which limits the statistical power. As the combination of spectroscopy, CEST and quantitative MRI required comparably long acquisition times, only tumor patients in relatively good clinical condition were able to take part in the study. Another limitation is the dependence on ^31^P spectroscopy, as it remains the only viable, noninvasive method for measuring pHi. The spatial resolution of ^31^P spectroscopy at 3 T is limited compared with higher field strength and consequently the higher resolution of our CEST and T1 data could not be fully exploited and partial volume effects, especially in CE, had to be accepted to some degree.

## 5 CONCLUSION

The commonly used APTw MTR_asym_ and MTR_Rex_ are strongly influenced by the T1‐relaxation time, which is typically elevated in tumor tissue compared with CNAWM. As the pH value is also highest in the tumor center, the correlation with both APT‐CEST metrics suffers from this general development, as the correlation of T1 and pHi in our data demonstrated. Thus, care must be taken when interpreting the APTw MTR_asym_ and MTR_Rex_ as pH weighted marker in tumor data.

Our real‐life data further imply that AREX is likely able to reflect pH_i_ changes in tissue as long as the concentrations of water and amide protons do not shift at the same time. Changes of these proton concentrations in pathologic tissue seemed to be relevant enough to limit the interpretability of AREX.

## Supporting information


**Figure S1** Scatter Plots with linear regression line and 95% confidence interval. R‐values from Pearson's correlation for the correlation of the intracellular pH levels measured by ^31^P‐MRS (pHi) and AREX for (a) T2 hyperintense tissue and (b) contrast enhancing tissue.
**Figure S2** Phantom experiment for the investigation of the pH sensitivity via APT‐CEST effect and ^31^P‐MRS. Phantom tubes were adjusted with the same amount on gelatin powder for providing a CEST effect within a PSA solution. Different T1 times and pH were adjusted for each tube by using Gadovist and KOH solution. (a) shows the acquired Z‐spectra of these 3 phantoms tubes with increasing saturation at 3.5 ppm resulting from increased pH and T1. (b) shows the relative shift of inorganic phosphate (Pi) as consequence of the pH variations. The red tube (1300ms) has the highest pH, indicated by its leftward shift in the phosphorus spectrum. The tube with 450ms T1 has the lowest pH, seen by its rightward spectral position. (c) shows the parametric maps read out at 3.5 ppm once for the APTw MTR_asym_ and for AREX across 8 slices. When isolating the amide signal only from further MT effects and particular from R2 of water by using the inverse Z‐spectrum, we receive the exchange‐dependent relaxation (Rex) as shown in (d). The data show that the APT effect at constant amide proton concentration increases with higher pH but also with increasing T1 time. To counteract the different longitudinal relation times of water, a T1 correction is performed according to the literature to obtain the apparent exchange‐dependent relaxation (AREX) as shown in (e). After the last correction, the signal depends only on pH. In the last sub‐figure (f) the calculated spectroscopic pH‐value is compared against the pH‐value from APT‐CEST.

## Data Availability

The data that support the findings of this study are available on request from the corresponding author. The data are not publicly available due to privacy or ethical restrictions.
