# Supplementary material for: Pathological tissue changes in brain tumors affect the pH‐sensitivity of the T1‐corrected apparent exchange dependent relaxation (AREX) of the amide protons
Source: NMR Biomed. 2024 Oct 28;38(1):e5285. doi: 10.1002/nbm.5285 (PMC11602268; doi:10.1002/nbm.5285)
Supplement: Supplementary file 1 — Figure S1 Scatter Plots with linear regression line and 95% confidence interval. R‐values from Pearson's correlation for the correlation of the intracellular pH levels measured by 31P‐MRS (pHi) and AREX for (a) T2 hyperintense tissue and (b) contrast enhancing tissue. Figure S2 Phantom experiment for the investigation of the pH sensitivity via APT‐CEST effect and 31P‐MRS. Phantom tubes were adjusted with the same amount on gelatin powder for providing a CEST effect within a PSA solution. Different T1 times and pH were adjusted for each tube by using Gadovist and KOH solution. (a) shows the acquired Z‐spectra of these 3 phantoms tubes with increasing saturation at 3.5 ppm resulting from increased pH and T1. (b) shows the relative shift of inorganic phosphate (Pi) as consequence of the pH variations. The red tube (1300ms) has the highest pH, indicated by its leftward shift in the phosphorus spectrum. The tube with 450ms T1 has the lowest pH, seen by its rightward spectral position. (c) shows the parametric maps read out at 3.5 ppm once for the APTw MTRasym and for AREX across 8 slices. When isolating the amide signal only from further MT effects and particular from R2 of water by using the inverse Z‐spectrum, we receive the exchange‐dependent relaxation (Rex) as shown in (d). The data show that the APT effect at constant amide proton concentration increases with higher pH but also with increasing T1 time. To counteract the different longitudinal relation times of water, a T1 correction is performed according to the literature to obtain the apparent exchange‐dependent relaxation (AREX) as shown in (e). After the last correction, the signal depends only on pH. In the last sub‐figure (f) the calculated spectroscopic pH‐value is compared against the pH‐value from APT‐CEST. [file NBM-38-e5285-s001.docx]

**Supplementary Material**

We demonstrate that various APT-CEST metrics exhibit a correlation with spectroscopic pHi. This observation is influenced by multiple MT effects, including aliphatic NOE and ssMT, as well as T1 relaxation time. These factors collectively enhance the correlation effect in tumor tissue, thereby potentially distorting the interpretation when relying solely on the Rex term.

Extending the analysis from Fig. 3, we inhere show the relationships in T2-H and CE-enhancing tissues for AREX (*Rex=ksw*fb)* and ^31^P-MRS derived pH_i_. In comparison to CNAWM, these tissue types demonstrate a minimal, negative correlation, which disturbs the overall correlation when all tissue types are considered (Fig.2 d). Due to the pronounced heterogeneity in tumors, there is likely a variation in the composition of protein/peptide concentrations as well as the water content, which leads to a deviation in fb and thus influences Rex, resulting in a diminished or non-existent correlation with pHi.


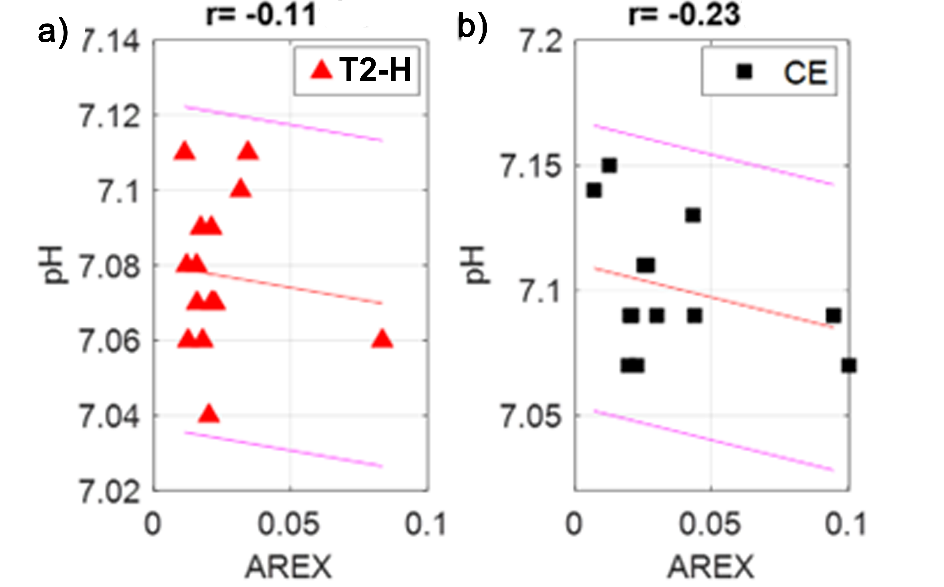


***Fig S1*** *Scatter Plots with linear regression line and 95% confidence interval. R-values from Pearson’s correlation for the correlation of the intracellular pH levels measured by* ^31^P *-MRS* (pH_i_) *and AREX for (a) T2 hyperintense tissue and (b) contrast enhancing tissue.*

When considering AREX in CNAWM only, we hypothesize that the tissue-specific properties remain relatively unchanged. Under this assumption, the proton fraction between water and amide protons can be considered a constant property, thus changes in AREX are dominated by the pH-dependent exchange rate term (Fig.3 c).

Assuming a constant amide proton concentration, but different T1 relaxation times, it could be shown in vitro that the apparent exchange-dependent relaxation (AREX), which corrects for different longitudinal relaxation times of water, correlates with pHi. fAs the T1 measurement was shown to be quite stable in vivo (average rescan error below 2% ^1^), we assume that the possible error of the T1 measurement in our in vitro experiment is neglectable.

The experiment comprises three phantom tubes, each containing an identical amount of gelatin for the APT-CEST effect, but calibrated to different pH values with KOH and to different longitudinal relaxation times with Gadovist. An adaption of T2 was neglected. The T1 times were adjusted to 450 ms, 850 ms and 1300 ms by giving different amounts of Gadovist solution to the phantom tubes.

This APT-CEST effect can be seen in the Z-spectra at 3.5 ppm by the saturation peak (a) and in the ^31^P-MRS spectra by the relative shift of the inorganic phosphate, suggesting changes in pH (b).When observing the parametric maps at +3.5 ppm as shown in (c) for the APTw MTRasym (contains further MT effects and the dependence of T1 on water) and the apparent exchange-dependent relaxation - AREX (isolated amide peak with T1-correction of water), the image contrast reveals an increased signal intensity for both metrics with rising pH as shown in the legend. If we consider MTR_Rex_ (d), which largely eliminates the APT signal from the overlying semi-solid MT by omitting R_eff_ and particular frees the signal of R_2_ from water, we observe an increase in signal that is dependent on both - the pH value itself and the T1 relaxation time of water. If the T1 dependence is further corrected via AREX (e), the signal change is solely due to the pH change, assuming the same amide proton concentration. In the subsequent conversion to pH values using AREX and comparison with the spectroscopically determined pH values, both data sets show a close relationship (f).

The spectroscopic pH values for tubes 1 to 3 were measured using modified Henderson-Hasselbalch equations [Eq.1] to be 7, 7.06 and 7.16, we assumed that the concentration ratio between water and amide protons is 0.0023 to match the pH value of 7 in the first tube. With this concentration ratio, the pH values calculated by APT CEST were 7, 7.08, 7.15.

The formula for calculating pH via CEST is given by:

pH = 6.5 + log10 (AREX(+3.5 ppm) / fs *5.57)

where fs is the proton fraction and 5.57 × 109 s−1 was obtained in the work of Zhou et al (2003)^2^ for the base-catalyzed exchange rate (k_base_). This indicates that AREX reflects the pH-sensitive exchange rate at a constant concentration ratio fb, which is also suggested by our in vivo data assuming similar conditions.


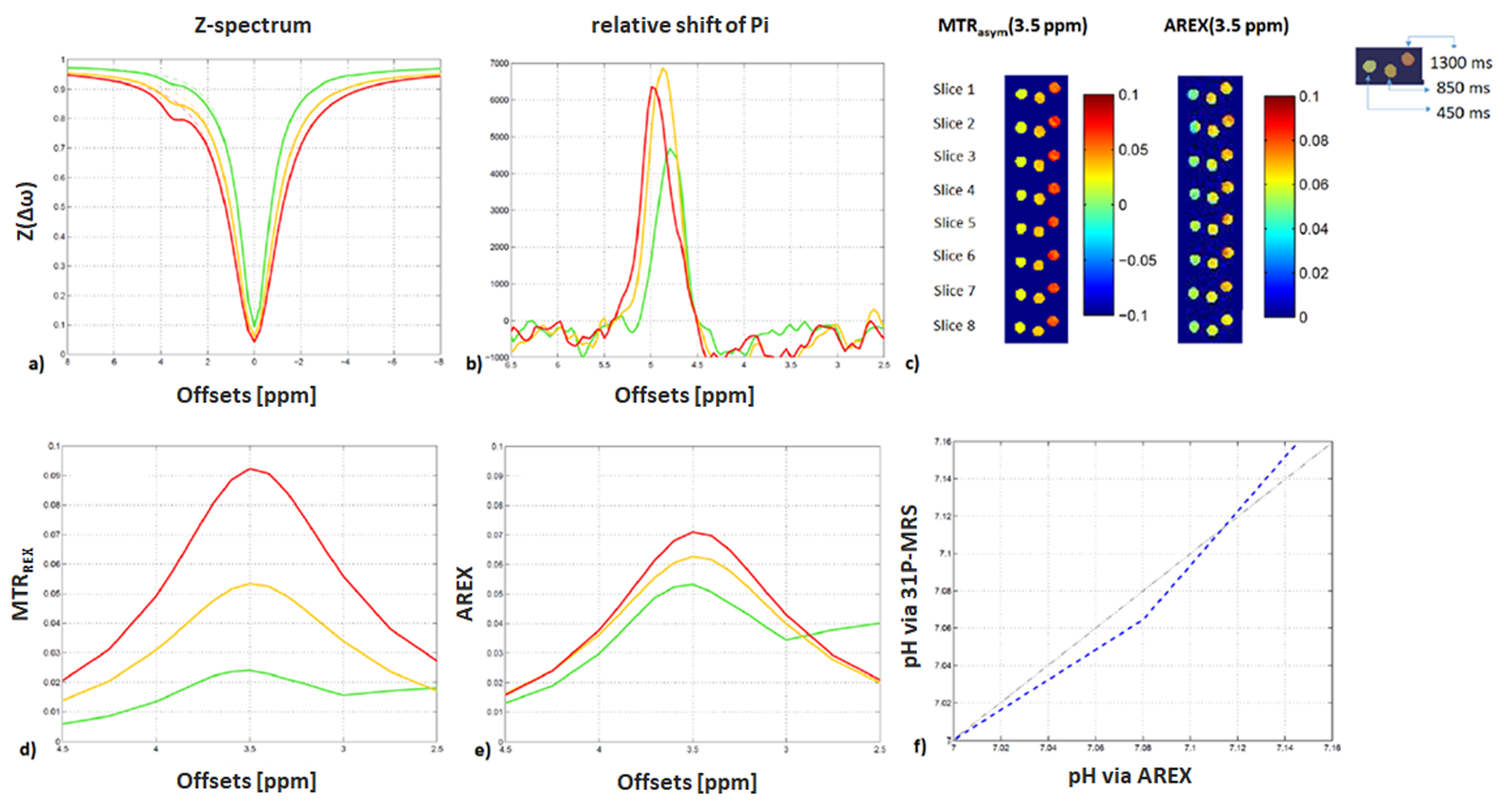


***Fig S2*** *Phantom experiment for the investigation of the pH sensitivity via APT-CEST effect and ^31^P-MRS. Phantom tubes were adjusted with the same amount on gelatin powder for providing a CEST effect within a PSA solution. Different T1 times and pH were adjusted for each tube by using Gadovist and KOH solution. (a) shows the acquired Z-spectra of these 3 phantoms tubes with increasing saturation at 3.5 ppm resulting from increased pH and T1. (b) shows the relative shift of inorganic phosphate (Pi) as consequence of the pH variations. The red tube (1300ms) has the highest pH, indicated by its leftward shift in the phosphorus spectrum. The tube with 450ms T1 has the lowest pH, seen by its rightward spectral position. (c) shows the parametric maps read out at 3.5 ppm once for the APTw MTR_asym_ and for AREX across 8 slices. When isolating the amide signal only from further MT effects and particular from R2 of water by using the inverse Z-spectrum, we receive the exchange-dependent relaxation (Rex) as shown in (d). The data show that the APT effect at constant amide proton concentration increases with higher pH but also with increasing T1 time. To counteract the different longitudinal relation times of water, a T1 correction is performed according to the literature to obtain the apparent* *exchange-dependent relaxation (AREX) as shown in (e). After the last correction, the signal depends only on pH. In the last sub-figure (f) the calculated spectroscopic pH-value is compared against the pH-value from APT-CEST.*

References

1. Gracien R-M, Maiworm M, Brüche N, et al. How stable is quantitative MRI? - Assessment of intra- and inter-scanner-model reproducibility using identical acquisition sequences and data analysis programs. *Neuroimage*. 2020;207:116364. doi:10.1016/j.neuroimage.2019.116364

2. Zhou J, Payen J-F, Wilson DA, Traystman RJ, van Zijl PCM. Using the amide proton signals of intracellular proteins and peptides to detect pH effects in MRI. *Nat Med*. 2003;9(8):1085-1090. doi:10.1038/nm907
